# The inhibitory effect of colloidal bismuth hydroxide gel on *Escherichia coli* O157:H7 and on the activity of Shiga toxins

**DOI:** 10.1186/1756-0500-7-875

**Published:** 2014-12-04

**Authors:** Tomás Subils, Cecilia Casabonne, Claudia Balagué

**Affiliations:** Faculty of Biochemistry and Pharmaceutical Sciences, Department of Clinical Bacteriology, National University of Rosario, Suipacha 531, S2002LRK Rosario, Santa Fe, Argentina

**Keywords:** *E. coli* O157:H7, Shiga toxin, Colloidal bismuth, Shigatoxigenic phage

## Abstract

**Background:**

Shiga toxin-producing *Escherichia coli* (STEC) is the causative agent of hemolytic uremic syndrome (HUS). Colloidal bismuth hydroxide gel (CBHG) is an anti-diarrheal and antisecretory compound, which does not inhibit gastrointestinal motility and reaches an in vivo gut concentration of 10.8 mg/ml of bismuth. Its action on bacteria has not been studied. We analyzed its inhibitory effects on STEC, as well as the deactivation of the Shiga toxin (Stx) and its ability to block the spread of genes encoding Stx. We determined a minimum inhibitory concentration and bactericidal concentration for the STEC O157:H7 strain (EDL933), with CBHG and Chobet® bismuth cream with pectin (CBCHP). We analyzed its effect on Stx by means of cytotoxicity assay and ELISA, as well as its effect on the free 933 W Stx phage.

**Results:**

Effect on the EDL933 strain: CBHG: MIC 10 mg/ml of bismuth. CBCHP: MIC 6 mg/ml and MBC 15 mg/ml of bismuth. Effect on EDL933 virulence factors: significant decrease in active Stx and 933 W Stx phage titer. ELISA did not find significant differences with treatment.

**Conclusions:**

The results obtained may be useful in the development of new therapeutic strategies based on the use of CBHG to prevent or improve the prognosis of HUS, as it can be used to control STEC infections.

## Background

Hemolytic uremic syndrome (HUS) is a clinical and anatomopathological entity characterized by the acute manifestation of kidney damage, microangiopathic hemolytic anemia and thrombocytopenia. It may also affect other parenchymas such as the intestine, pancreas, heart and central nervous system [1].

Shiga toxin-producing *Escherichia coli* (STEC) is reported to be the most significant causative agent of HUS, whereas *E. coli* 0157:H7 is the prevalent serotype. STEC has been identified as one of the emerging pathogens with the greatest impact on foodborne infections. Although these pathogenic strains produce several virulence factors, the main virulence factor is the Shiga toxin (Stx), an A-B-type toxin that inhibits protein synthesis in target cells. The Stx produced by STEC in the intestine are believed to enter the systemic circulation and cause damage to distant organs, especially the kidneys. Some studies suggest that the risk of serious complications in STEC infection, such as HUS, is related to the presence and quantity of Stx produced during the infection [2]. There are two main types of Stx in these strains, encoded on the *stx1* and *stx2* genes, which have a 56% homology, and each of them has more than one closely related variant [3]. Other genes may contribute to the virulence of the aforementioned strains, particularly the *eae* gene, which encodes the intimin protein that facilitates the binding of the bacterium to epithelium of the intestine; and an enterohemolysin (gene *ehxA*) [4, 5].

Both *E. coli* O157:H7 and the Stx-encoding phages or bacteriophages play a prominent role in the spread of virulence genes between different species of bacteria, in hosts and in the environment [6].

In Argentina, HUS is endemic and is the main cause of pediatric acute kidney failure [7] and the second cause of chronic kidney failure, making it responsible for 20% of transplants in children and adolescents [8]. Some studies have found that the STEC strains isolated in Argentina are similar to the reference STEC strain EDL933 [9]. To date, a specific treatment for HUS has not yet been developed, and some data have shown that the use of antibiotics in infected children increases the risk of developing the disease [10]. The exposure of *E. coli* O157:H7 strains to antibiotics can lead to the increased spread of phages and Stx production [11, 12].

In Argentina, Chobet® bismuth cream with pectin (CBCHP) has been prescribed as an anti-diarrheal drug for oral use for over 60 years. CBCHP does not inhibit intestinal motility and contains 30 mg/ml of colloidal bismuth hydroxide gel (CBHG) and pectin as active ingredients. Furthermore, bismuth compounds have been used extensively in gastroenterology. Previous studies in patients found bismuth absorption in the large intestine of below 1% and a theoretical concentration of 10.8 mg/ml [13]. Tests have also been done on the interaction of bismuth salts with fruit juices, ascorbic acid and thiols for the purpose of producing active, soluble bismuth products. The interaction of salts with thiol-containing molecules is due to the high affinity of the bismuth ion for sulfhydryl groups [14]. The antibacterial activity of the bismuth ion is lower as an inorganic salt and requires high concentrations. Some studies suggest that this activity can be increased by the use of lipophilic thiol agents [15]. In this regard, the use of CBHG has been demonstrated to generate antibacterial activity without the need for high concentrations.

Many epidemiological studies have confirmed the efficacy of treatments with bismuth compounds for the prevention of traveler’s diarrhea, particularly for the various virotypes of *E. coli, Salmonella spp.* and *Shigella spp.* [16, 17]; for the treatment of acute diarrhea from rotavirus and enterotoxigenic *E. coli* in children [18–20]; and as a supplement in the treatment of gastric and duodenal ulcers from *Helicobacter pylori* [21], among other applications.

Despite this vast and extensive background in the use of bismuth compounds for the prevention and treatment of diarrhea, there is still controversy regarding their complete mechanism of action. Some studies have indicated that bismuth compounds inhibit the intestinal secretion caused by *Vibrio cholerae* and enterotoxigenic *E. coli* toxins [22] and decrease the cell invasion of enteroinvasive *E. coli* [23]. Also, bismuth enhances the opsonophagocytosis of *Klebsiella pneumoniae*, thereby reducing the expression of the capsule [24], and reversibly represses the expression of fimbriae in enteropathogenic and uropathogenic *E. coli* strains [25]. A study by Brogan *et al.* [26] showed dithiol bismuth to be an inhibitor of the Rho protein of *E. coli,* an essential protein that controls the expression of several genes in Gram-negative bacteria. Thus, the inhibitory and antibacterial activity of bismuth compounds is believed to be the result of multiple mechanisms.

The aim of this study was to seek potential therapeutic strategies for the prevention and treatment of HUS, studying the effect of CBHG in clinical and subclinical concentrations on the viability and on the principal pathogenicity factors of a reference STEC strain.

## Methods

### Bacterial strain and bismuth compounds

We studied Chobet® bismuth cream with pectin (CBCHP) (Soubeiran Chobet, S.R.L., City of Buenos Aires, Argentina) because this is the only medicinal specialty with colloidal bismuth hydroxide gel (CBHG) as active ingredient with a concentration equivalent to 30 mg/ml of metallic bismuth. We used *E. coli* O157:H7 reference strain ATCC 43895 (EDL933) characterized by PCR for the presence of O157, *stx*_1_ and *stx*_2_.

### Determination of sensitivity to bismuth compounds

We determined the minimum inhibitory concentration (MIC) and minimum bactericidal concentration (MBC) of CBHG and CBCHP in Mueller-Hinton broth of the strains studied [27, 28]. We started from 0.5 McFarland bacterial suspension. For the MIC test, MH-agar plates with different concentrations of bismuth were seeded by drop. We worked with dilutions of 1/50, 1/25, 1/15, 1/10, 1/5 and 1/3, which correspond to bismuth concentrations of 0.6, 1.2, 2, 3, 6 and 10 mg/ml, respectively. Meanwhile, in the case of MBC, MH-broth tubes with serial dilutions of bismuth were inoculated, incubated overnight at 37°C and grown in MH-agar plates in absence of bismuth. The dilutions used were 1/32, 1/16, 1/8, 1/4 and 1/2, which are equivalent to bismuth concentrations of 0.94, 1.88, 3.75, 7.5 and 15 mg/ml, respectively.

### Determination of bacterial attachment to the bismuth suspension

The bacterial culture (10^10^ bacteria/ml) was incubated with and without bismuth compounds at a subinhibitory concentration (2 mg/ml) in LB medium at 37°C for 18 hours with shaking. Samples were then collected at specific time intervals (6, 24 and 168 hours) to determine the total number of viable microorganisms, while other samples were centrifuged at 1000 rpm for 5 minutes. Supernatant bacteria were considered not attached and they were counted on agar plates [28]. The number of attached bacteria was obtained by subtracting the number of not attached bacteria from the total viable bacteria.

### Effect of Stx expression according to cytotoxicity assay

The *E. coli* O157:H7 EDL933 strain was cultivated in LB medium at 37°C for 18 hours with shaking. The bacterial culture was diluted in 5 ml of LB broth together with the bismuth compounds to be analyzed: CBHG in dilutions of 1/5 and 1/3, which correspond to bismuth concentrations of 6 and 10 mg/ml, respectively; and CBCHP in dilutions of 1/10 and 1/3, which are equivalent to bismuth concentrations of 3 and 10 mg/ml, respectively. They were then incubated for 18 hours at 37°C with stirring at 140 rpm. We performed a positive control without bismuth compounds, negative controls without bacterial culture for each compound and concentration used, as well as a double negative control without bismuth compounds or bacterial culture. The cultures were centrifuged at 8,000 rpm for 10 minutes at 4°C, whereas the supernatants were separated, filtered through a 0.43 μm membrane and the bacterial presence controlled in MH-agar [29].

We used the technique of 96-well microplates seeded by co-culture of Vero cells with the samples [30]. The Vero cells grew in a minimum essential medium (MEM, Sigma 0643) (with 200 mg/l of streptomycin and 100 mg/l of penicillin), to which 10% fetal bovine serum (FBS) was added at 37°C with 5% of CO_2_. The cells were detached using trypsin and were transferred to an MEM without SFB. We inoculated 100 μl of a 2.5 × 10^6^ cells/ml suspension in each of the microplate’s wells.

Next, we seeded the samples of the supernatants from the bacterial cultures and made dilutions (1, 1/2, 1/4, 1/8 and 1/16) with MEM with 15% FBS. The microplates were incubated at 37°C with 5% of CO_2_, and readings were taken at 48 h with an inverted microscope. The percent cytotoxicity is an estimate of the cell monolayer destruction. We then determined the titer of 50% cytotoxic activity (DC50). The cells were stained and fixed with 0.75% violet crystal solution in 40% methanol [31].

### Determination of Stx concentration by means of enzyme immunoassay

We used the RIDASCREEN® Verotoxin (R5701, R-Biopharm Latinoamérica S.A., City of Buenos Aires, Argentina) test to detect Stx1 and Stx2. Following the manufacturer’s specifications, we assayed the supernatants of the bacterial cultures treated 18 h with CBHG (6 mg/ml) to determine extracellular Stx. Measurements were taken photometrically with a wavelength of 450 nm. The average was calculated and compared with untreated bacterial cultures.

### Effect on free phage 933 W

We analyzed the effect of CBHG and CBCHP on the Stx-encoding lambda 933 W phage. We titered and incubated the phage suspension with the aforementioned compounds at four different dilutions: 1/24, 1/12, 1/6 and 1/3, which correspond to bismuth concentrations of 1.25, 2.5, 5 and 10 mg/ml, respectively, for 24 hours at 37°C with continuous shaking. Later, we confirmed the presence of the phages by means of the double-agar layer technique, using *E. coli* DH5α as the indicator strain and we determined the number of lysis plates formed (plaque forming units - PFU/ml) [32].

### Statistical analysis

All tests were performed independently five times, and the data were statistically evaluated by variance analysis, followed by the Tukey-Kramer multiple comparison test.

## Results

### Study of the effect on STEC

When we performed the inhibition assays with CBHG, we observed an MIC corresponding to 10 mg/ml of bismuth for the reference STEC strain. However, MBC was not detected for the doses studied. In the case of CBCHP, we observed an MIC corresponding to 6 mg/ml of bismuth for the STEC and a MBC corresponding to 15 mg/ml of bismuth. CBHG exhibited a greater degree of bacterial attachment (p < 0.05) than CBCHP. At the same time, CBHG attachment remained almost constant over time (about 90 to 98%). With CBCHP, we observed a statistically significant increase (p < 0.05) in the percentage (from 22.9 to 41.1%) of cells attached from 6 to 24 h, and then the attachment rate leveled out (Table 1). No significant difference was observed in the number of viable microorganisms attached without bismuth compounds, under the conditions applied.

**Table 1:** **MIC, MBC and percentage of bacterial attachment to CBHG and CBCHP**

Bacterial strains	Concentration of bismuth (mg/ml)				Colony forming units attached (%)					
	CBHG ^1^		CBCHP ^2^		CBHG ^1^			CBCHP ^2^		
	MIC ^3^	MBC ^4^	MIC ^3^	MBC ^4^	Incubation time			Incubation time		
					6 h	24 h	168 h	6 h	24 h	168 h
STEC EDL933	10	Higher than the dose	6	15	89.6*	94.1*	97.7*	22.9	41.1*	41.4*

*p < 0.05, ^1^CBHG: colloidal bismuth hydroxide gel; ^2^CBCHP: Chobet® bismuth cream with pectin. ^3^MIC: minimum inhibitory concentration; ^4^MBC: minimum bactericidal concentration.

### Study of the effect on pathogenicity factors of *E. coli*O157:H7

**Figure 1:**
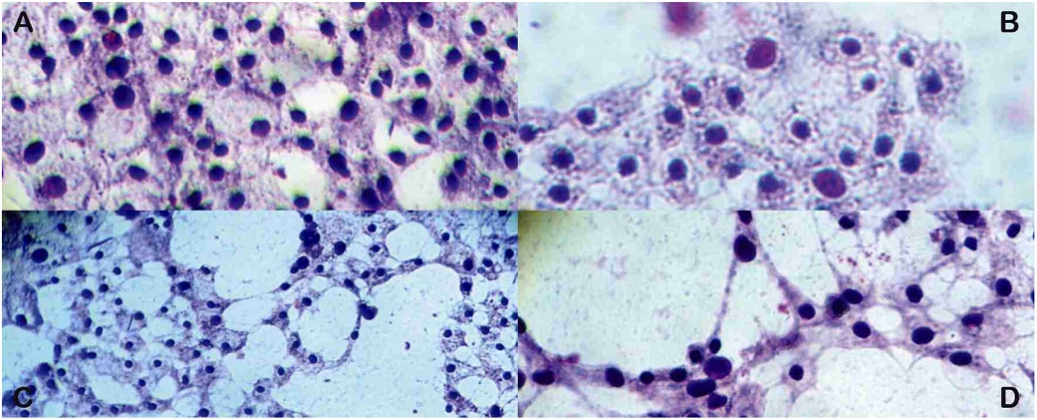
**Microscopy of monolayers of Vero cells. A**. Normal monolayer, 1/32 CBHG 3 mg/ml. **B**. With 20% cytotoxicity, 1/4 CBCHP 3 mg/ml. **C**. With 50% cytotoxicity 1/16 CBHG 3 mg/ml. **D**. With 80% cytotoxicity 1/2 CBHG 3 mg/ml.

We observed a significant reduction (p < 0.05) in the active Stx titer in the samples treated with a 1/5 dilution of CBHG, which corresponds to a bismuth concentration of 6 mg/ml; and with a 1/10 dilution of CBCHP, which is equivalent to a bismuth concentration of 3 mg/ml. These results show that concentrations even lower than the MIC could inhibit Stx activity. However, no significant differences were observed at lower concentrations (Table 2). The following changes were observed by means of optical microscopy (Figure 1): brightness and progressive detachment of cells from monolayer, formation of cell surface in folds with multiple blebs, and finally release of several membrane-bound apoptotic bodies. The apoptotic cells were characterized by condensed chromatin, intensive nuclear fragmentation, cytoplasmic organelle disruption, formation of apoptotic bodies and cell shrinkage [33].

**Table 2:** **Effect of CBHG and CBCHP on Stx in Vero cells, expressed as cytotoxicity percentages**

Dilutions of toxins	Percentage of cytotoxicity (%)				
	Without bismuth (Control +)	CBHG ^1^		CBCHP ^2^	
		6 mg/ml	3 mg/ml	3 mg/ml	1.5 mg/ml
1/2	80	20	80	50^*a*^ *	100
1/4	80	-	80	20	50^*a*^
1/8	50^*a*^	-	60	-	50
1/16	50	-	50^*a*^	-	-
1/32	-	-	-	-	-

^*a*^50% cytotoxicity dose (CD_50_), *p < 0.05, ^1^CBHG: colloidal bismuth hydroxide gel; ^2^CBCHP: Chobet® bismuth cream with pectin.

However, when quantifying free Stx on supernatants, the enzyme immunoassay test yielded no significant differences (p = 0.6849) between the supernatant treated with bismuth and the untreated control at the dilutions studied.

**Figure 2:**
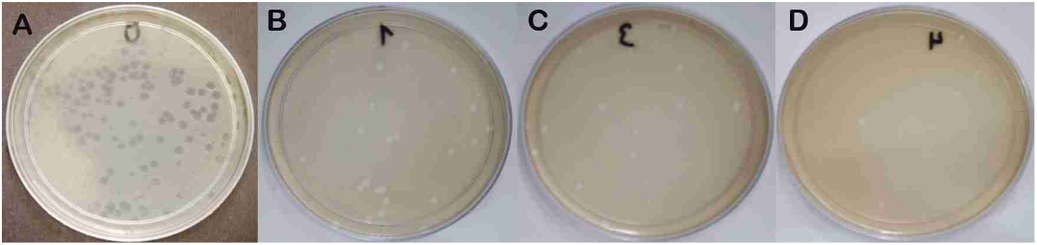
**Photographs of incubated phage lysis plates. A**. Without bismuth. **B**. With 1.25 mg/ml of bismuth. **C**. With 5 mg/ml of bismuth. **D**. With 10 mg/ml of bismuth.

As for the effect of the compounds on the Stx 933 W-encoding phage, a statistically significant decrease (p < 0.05) was observed in the titer of the phage treated with both CBHG and CBCHP in comparison with the untreated titer. The decrease in the titer of the phage was 90% for a CBHG dilution of 1/3, which corresponds to a bismuth concentration of 10 mg/ml; and 86% for a CBHG dilution of 1/6, which is equivalent to a bismuth concentration of 5 mg/ml. In the tests performed with CBCHP, the decrease in the titer of the phage was 82% for a CBCHP dilution of 1/3, which corresponds to a bismuth concentration of 10 mg/ml; and 73% for a CBCHP dilution of 1/6, which is equivalent to a bismuth concentration of 5 mg/ml (Table 3 and Figure 2).

**Table 3:** **Effect of CBHG and CBCHP on the 933 W Shiga toxin-encoding phage**

Bismuth concentration (mg/ml)	Phage titer (PFU/ml)	Percentage of reduction (%)	Phage titer (PFU/ml)	Percentage of reduction (%)
0	2.6 × 10^3^ ± 6.1 × 10^2^		3.9 × 10^3^ ± 2.1 × 10^2^	
	CBCHP^1^		CBHG^2^	
1.25	1.1 × 10^3^ ± 4.9 × 10^2^*	48	9.3 × 10^2^ ± 2.5 × 10^2^**	76
2.5	1.0 × 10^3^ ± 3.9 × 10^2^*	56	7.4 × 10^2^ ± 1.3 × 10^2^**	80
5	7.2 × 10^2^ ± 1.1 × 10^2^*	73	4.8 × 10^2^ ± 5.0 × 10^1^**	86
10	4.2 × 10^2^ ± 2.0 × 10^2^*	82	3.8 × 10^2^ ± 6.0 × 10^1^**	90

*P < 0.05, **P < 0.001, results expressed as the mean ± the SD of five experiments.

^1^CBCHP: Chobet® Bismuth Cream with Pectin, ^2^CBHG: colloidal bismuth hydroxide gel.

## Discussion

As a result of the anti-diarrheal activity exhibited by bismuth compounds in the treatment of various pathologies associated with infections of the digestive apparatus in humans, the possible mechanisms of action of these compounds have been studied with renewed interest. In choosing CBHG as our object of study, we considered the following advantages: a) the bismuth in CBHG is in a colloidal state; b) the bismuth in CBHG does not appear in the form of salt, such as salicylate, subsalicylate or subgallate; c) the medicinal specialty CBCHP is an anti-diarrheal drug for oral use suitable for administration in both children and adults, which was developed in Argentina and has been on the market for over 60 years at a low cost.

We based our study on the analysis of the effect of CBHG and of CBCHP in differing concentrations, all within the usual approved dosage, on STEC strains and their virulence factors.

First, we assessed the sensitivity of STEC to CBHG and CBCHP. The MIC is the most commonly used indicator in choosing an antimicrobial therapy. In all cases tested, MIC values were within the order of bismuth concentrations of 6 and 10 mg/ml. The standard concentration of bismuth both in CBHG and in CBCHP is 30 mg/ml, three to five times higher than the MICs found. Therefore, STEC O157:H7 is sensitive to treatment with CBHG at lower concentrations than those usually used in therapeutics and at the concentrations reached in vivo in the gut (10.8 mg/ml of bismuth). The MIC and MBC values obtained show higher sensitivity to CBCHP.

Bacterial resistance is a natural biological phenomenon whose most serious consequence is the failure of antimicrobial therapy, with the resulting increase in morbimortality and in the cost of treatment. To date, it has not been possible to find any publication providing data on bacterial resistance to bismuth.

Both CBHG and the CBCHP have demonstrated their ability to attach to and drag the STEC studied. CBHG exhibited a greater ability to attach to bacterial cells and drag them with slight centrifugation. The virulence of the bacterial strains is directly related to their ability to attach to the gastrointestinal epithelial walls [34]. The mechanical attachment to the used compounds would obstruct the bacterial attachment to the gastrointestinal epithelium, preventing colonization and favoring the rapid expulsion of pathogens outside the tract.

Furthermore, the data obtained show a significant reduction in the cytotoxic activity of Stx on Vero cells with subinhibitory and subclinical concentrations of both CBHG and CBCHP. No significant differences (p < 0.05) were observed compared to controls with regard to the quantification of toxins by immunoadsorption. Therefore, we suggest that the decrease in the activity of Stx is the result of the direct interaction of CBHG with the activity of toxins on eukaryotic cells.

The activity of Stx is directly related to the enzymatic function of the cytotoxic subunit A [35]. Also, the association of the toxin with severe systemic pathologies implies differences in the pentameric subunit B and in its binding affinity to the Gb3 receptor in eukaryotic cells [36]. Although some compounds can act as neutralizers in the binding process of the toxin to its receptor, thus generating inhibition of cytotoxicity on Vero cells [37], we consider that the effect observed may be related to the fixation of bismuth on one or more cysteine-rich Stx sites involved in cytotoxicity [38, 39].

The results obtained in tests with CBHG and CBCHP on the free bacteriophage are very promising, with reductions of 80 and 90% in the phage titer. Stx damages endothelial and renal tubular cells, and is therefore considered a major virulence factor [40]. Stx1 and Stx2 are encoded in the lambdoid prophage genome, and release phage particles which infect other neighboring cells or which remain in the medium as potential vehicles of virulence genes [35]. The deactivation of the free bacteriophage, caused by CBHG and by CBCHP, would inhibit the spread of Stx-encoding genes to other uninfected cells, which in turn would prevent the generation of new Stx-producing strains.

## Conclusions

We believe that the results obtained in this study are very promising from a therapeutic point of view in the prognosis of HUS, and that the possibility of using CBHG as a preventive measure should be assessed in the event of diagnosis or suspicion of infection by *E. coli* O157:H7 or other Shiga toxigenic strains such as the recently emerging serotype O104:H4.
